# Correction: Time series models for prediction of leptospirosis in different climate zones in Sri Lanka

**DOI:** 10.1371/journal.pone.0318175

**Published:** 2025-01-23

**Authors:** The PLOS ONE Staff

## Notice of republication

This article was republished on January 13, 2025, to correct an error in the affiliation 1 and the author list: Rupika Abeynayake was erroneously displayed as Rupika Abeynayake R. The publisher apologizes for the errors. Please download this article again to view the correct version. The originally published, uncorrected article and the republished, corrected articles are provided here for reference.

## Supporting information

S1 File Originally published, uncorrected article. (PDF)

S2 File Republished, corrected article. (PDF)
